# Personalized Medicine Based on Theranostic Radioiodine Molecular Imaging for Differentiated Thyroid Cancer

**DOI:** 10.1155/2016/1680464

**Published:** 2016-04-28

**Authors:** Byeong-Cheol Ahn

**Affiliations:** Department of Nuclear Medicine, Kyungpook National University School of Medicine and Hospital, 50 Samduk-dong 2-ga, Jung-gu, Daegu 700-721, Republic of Korea

## Abstract

Molecular imaging based personalized therapy has been a fascinating concept for individualized therapeutic strategy, which is able to attain the highest efficacy and reduce adverse effects in certain patients. Theranostics, which integrates diagnostic testing to detect molecular targets for particular therapeutic modalities, is one of the key technologies that contribute to the success of personalized medicine. Although the term “theranostics” was used after the second millennium, its basic principle was applied more than 70 years ago in the field of thyroidology with radioiodine molecular imaging. Differentiated thyroid cancer, which arises from follicular cells in the thyroid, is the most common endocrine malignancy, and theranostic radioiodine has been successfully applied to diagnose and treat differentiated thyroid cancer, the applications of which were included in the guidelines published by various thyroid or nuclear medicine societies. Through better pathophysiologic understanding of thyroid cancer and advancements in nuclear technologies, theranostic radioiodine contributes more to modern tailored personalized management by providing high therapeutic effect and by avoiding significant adverse effects in differentiated thyroid cancer. This review details the inception of theranostic radioiodine and recent radioiodine applications for differentiated thyroid cancer management as a prototype of personalized medicine based on molecular imaging.

## 1. Introduction

The term “theranostics” is portmanteau word of “therapeutics” and “diagnostics” and was coined by John Funkhouser (Chief Executive Officer of PharmaNetics) in 2002 to describe his company's business model for developing diagnostic tests directly linked to the application of specific therapies [1, 2]. As an invaluable tool in personalized medicine, theranostics can be defined as a diagnostic methodology for individually tailored therapeutic intervention, and it customizes healthcare practices to an individual patient by eliminating unnecessary treatments for patients whom a standard therapy is not appropriate and/or by optimizing a therapeutic plan for a particular patient [3]. The theranostic system integrates diagnostic testing to detect the presence of a molecular target for which a specific therapeutic modality is intended [4].

Although we began using the term “theranostics” after the second millennium, the basic principle of theranostics has been applied for some time in the field of thyroidology; radioiodine was used as the first theranostic agent [5]. Radioiodine theranostics is a typical example of personalized medicine and has been used extensively for the management of differentiated thyroid cancer [6, 7]. Better understanding of thyroid cancer pathophysiology and advancements in bioengineering, electrical engineering, and radiochemical technologies have improved radioiodine theranostics, which contributes to a tailored personalized management system for differentiated thyroid cancer.

This review details the inception of theranostic radioiodine as well as recent updates in radioiodine applications for differentiated thyroid cancer, which is a prototype of personalized medicine based on molecular imaging.

## 2. Radioiodine and Differentiated Thyroid Cancer

Although radioiodine has been used to diagnose and treat differentiated thyroid cancer for more than 70 years, the accumulation of radioiodine in cancer cells was not fully understood until 1996, when the sodium iodide symporter (NIS) was first cloned by Carrasco et al. [8]. The NIS is an intrinsic plasma membrane glycoprotein with 13 transmembrane domains which actively transports one iodide into the cytosol of benign or malignant thyroid cells from extracellular fluid along with two sodium ions [9]. Administration of beta emitting radioiodine I-131 instead of naturally occurring stable iodine I-127 can selectively harm or kill the differentiated thyroid cancer cells that specifically accumulate iodine; in addition, the cells also can be imaged with gamma camera using gamma rays concomitantly emitted from I-131.

I-131 administration after total or near-total thyroidectomy can potentially have a tumoricidal effect on thyroid cancer cells that may persist after surgery, and radioiodine imaging using a gamma camera obtained at the time visualizes previously undiagnosed regional or distant metastatic lesions. In addition, postoperative I-131 administration may facilitate the early detection of a recurrence, based on serum thyroglobulin measurement or radioiodine imaging by removing residual normal thyroid tissues [10, 11]. Mazzaferri et al. had reported beneficial effects of I-131 administration as an initial therapy in differentiated thyroid cancer with long-term large cohort studies and ended up the debate of applying the I-131 administration to the disease which had spanned decades due to good prognosis and long course of the disease [12–14]. A number of subsequent studies confirmed the beneficial effects of reducing the recurrence and mortality rates and supported the use of I-131 administration as an initial adjuvant therapy for the disease [10, 11]. However, the beneficial effects of I-131 administration were not observed in all patients with the disease, and similar studies for patients at a lowest risk of recurrence or mortality revealed no such effect [10]. Therefore, individualized clinical decision-making is needed in certain patients who belong to subgroups in which this beneficial effect of I-131 administration is observed [10, 15–17].

## 3. Theranostic Molecular Imaging of Radioiodine

Radioiodine imaging was first molecular imaging performed by Dr. Benedict Cassen in 1950 at UCLA. He developed the rectilinear scanner and successfully imaged the gland, revealing biologic characteristics of the thyroid tissues using radioiodine before the era of tomographic imaging [18]. Molecular imaging allows visual representation, characterization, and quantification of the biological characteristics of cell and tissues within intact living organisms. Therefore, it can visualize the therapeutic targets of certain diseases [19].

**Figure 1:**
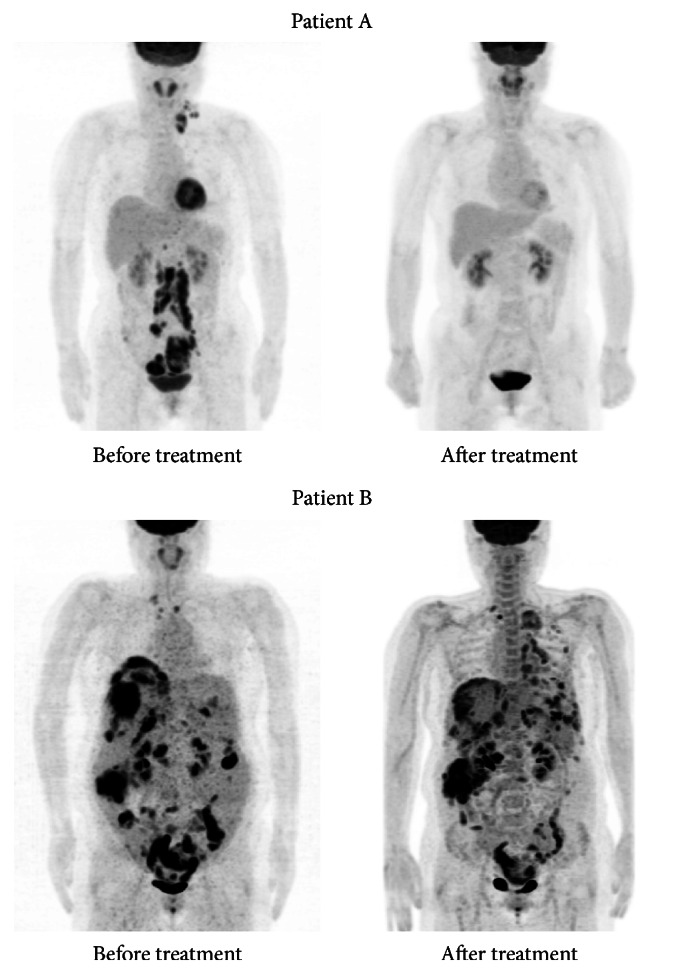
Patients A and B were diagnosed with advanced ovarian cancer. Pretreatment F-18 FDG PET imaging successfully visualized multiple cancerous lesions in the neck and abdominal cavity. However, the imaging was not able to predict the therapeutic response to the subsequent chemotherapy. Patient A achieved complete remission after chemotherapy; however, patient B progressed to disease status after chemotherapy.

In order to evaluate suspected but unproven cancers, diverse diagnostic imaging techniques have been applied, and these techniques can visualize and localize hidden cancerous lesions.  Figure 1 demonstrates F-18 FDG PET imaging of ovarian cancer as a typical example of diagnostic imaging. The F-18 FDG PET study successfully visualized hidden malignant foci; however, the study cannot predict therapeutic response to chemotherapy. Patient A received systemic chemotherapy due to far advanced disease stage based on the diagnostic imaging and complete remission was obtained by chemotherapy. However, patient B with a similar clinical condition as observed using diagnostic imaging underwent chemotherapy that did not yield any effects, based on the imaging, which was not able to forecast the therapeutic response, and the patient might experience adverse effects related to the chemotherapy.

**Figure 2:**
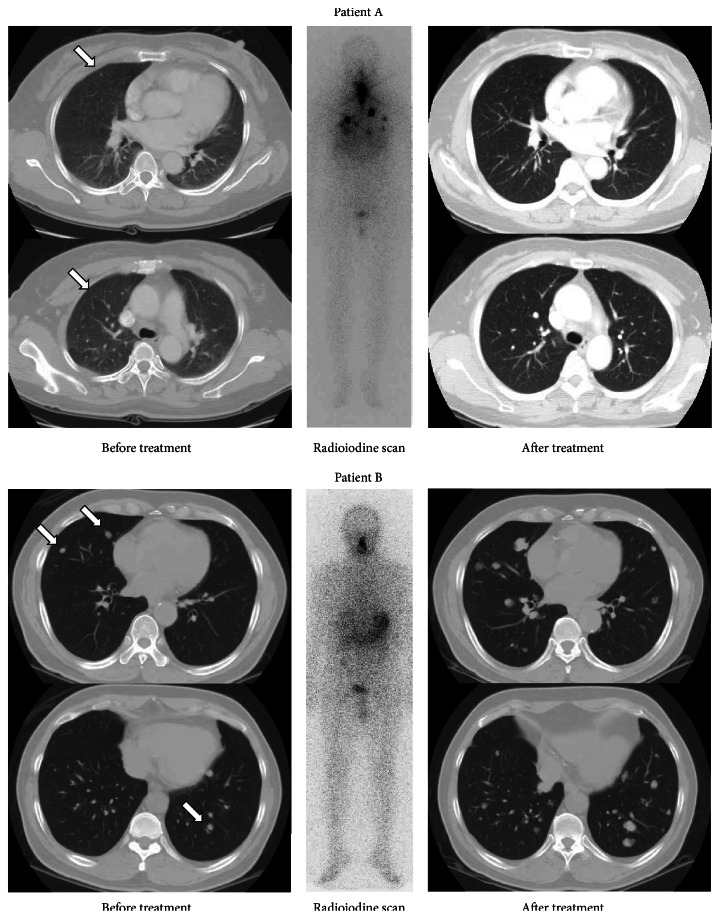
Patients A and B were diagnosed with thyroid cancer with pulmonary metastases (indicated by arrows) by pretreatment diagnostic CT imaging. Radioiodine imaging was able to forecast the therapeutic response to I-131 treatment. Patient A showed obvious uptake on radioiodine imaging and achieved complete remission after three I-131 treatments; however, patient B showed no radioiodine uptake on the imaging and progressed to disease status after I-131 treatment.

Unlike diagnostic imaging studies, radioiodine imaging can forecast response to therapy and can therefore be used for theranostic imaging, which can potentially alter the decision to treat with I-131 and finalize the subsequent therapeutic dose of I-131 [10].  Figure 2 demonstrates the benefit of theranostic radioiodine imaging, which is able to forecast therapeutic response of metastatic thyroid cancer to I-131 treatment. Patient A, who has radioiodine avid metastatic lesions on radioiodine imaging, received I-131 treatment three times and was disease-free after the I-131 treatments. Patient B, who has a radioiodine nonavid metastatic lesions on radioiodine imaging, revealed progressive disease after I-131 treatment.

To attain the best outcome, a therapeutic plan must be tailored to individual patients and can even be tailored to individual lesions in a patient. Theranostic radioiodine imaging can provide detailed biological status for each cancerous lesion and helps in predicting the therapeutic response of every lesion to I-131 treatment. Based on the imaging, ineffective I-131 treatment can be avoided, and furthermore, a lesion-based therapeutic plan can be established to overcome cancer heterogeneity, which is one of major therapeutic hurdles in oncologic diseases.

By applying dosimetric analysis to theranostic radioiodine imaging, the proper I-131 dose can be calculated and the response of each lesion to the I-131 therapy can be predicted by a lesion-based absorbed dose. The lesions might not respond completely to the therapy and may require a second-line treatment plan, such as surgical resection, external radiotherapy, or additional I-131 therapy to eradicate all lesions from individual patients. I-131 therapy combined with theranostic radioiodine imaging, which guides personalized or lesion-based therapeutic strategy, might be a representative model that demonstrates the ideal theranostic approach for personalized medicine.

**Figure 3:**
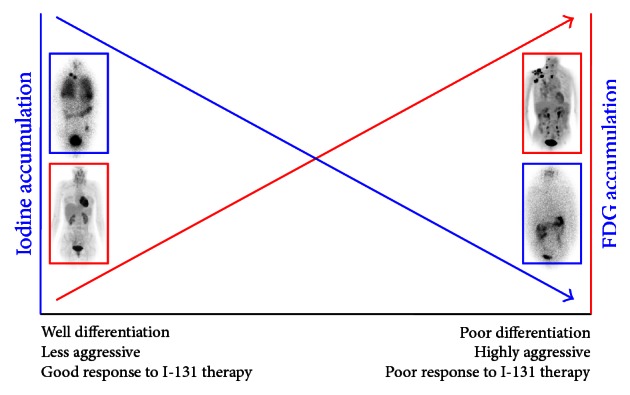
Flip-flop phenomenon of iodine and glucose avidity in thyroid cancer. Well-differentiated thyroid cancer has characteristics of the originating thyroid follicular cells, and therefore, the cancer cells can take up iodine, but not glucose. Therefore, the cancer lesions can be visualized on radioiodine imaging (blue box) but not on F-18 FDG PET imaging (red box). In contrast, poorly differentiated thyroid cancer does not have the characteristics of the originating thyroid follicular cells and has cancer hallmarks, and therefore, the cancer can take up glucose, but not iodine. Therefore, the cancer lesions can be visualized on F-18 FDG PET imaging, but not on radioiodine imaging.

I-131 treatment is the most important therapeutic option for treatment of differentiated thyroid cancer patients with iodine avid metastases, which cannot be resected by surgery [11]. Metastatic thyroid cancer lesions having iodine avidity are considered a more differentiated phenotype. However, metastatic lesions without iodine avidity, which do not respond to I-131 treatment, are a less differentiated phenotype and are prone to high glycolytic rates, which results in high glucose uptake on F-18 FDG PET. The inverse relation between iodine and glucose utilization in thyroid cancer according to the degree of differentiation, that is, the so-called flip-flop phenomenon, can occur in a thyroid cancer patient with multiple lesions due to heterogeneity of the metastases, which results in a mixed response to I-131 therapy [7, 20, 21]. Even though iodine avid metastases of a certain patient can be eradicated with I-131 therapy, FDG avid but iodine nonavid lesions of the patient need additional therapeutic strategies. Multimodal nuclear imaging is essential to design the lesion-based multimodal treatment strategy for patients with multiple heterogeneous metastatic lesions [22] (Figure 3).

## 4. Optimization of Personalized Medicine with Theranostic Radioiodine Imaging

Although I-131 therapy is one of the well-established standard therapeutic modalities in differentiated thyroid cancer and dosimetric determination of I-131 dose has theoretical advantages over empirical dose determination, the I-131 dose was almost always decided empirically on the basis of local clinical experiences or according to reference values reported in the literatures [11, 23]. Popularity of empirical dose determination is mainly related to the fact that dosimetric methods were not feasibly applicable with commercially available imaging instruments and workstations. However, in order to decide the optimal therapeutic dose of I-131, which can attain the therapeutic goal, and to preclude unnecessary side effects, individual dosimetry with a diagnostic I-131 dose is essential. Contrary to the empirical fixed dose method, the dosimetric method takes into account anatomical and I-131 biokinetic characteristics of cancerous lesions in each individual patient [23]. In addition, dosimetry can also be performed with post-I-131-treatment imaging, and it not only predicts the response to the treatment but also is helpful in developing the following diagnostic and therapeutic plans for each individual patient.

**Figure 4:**
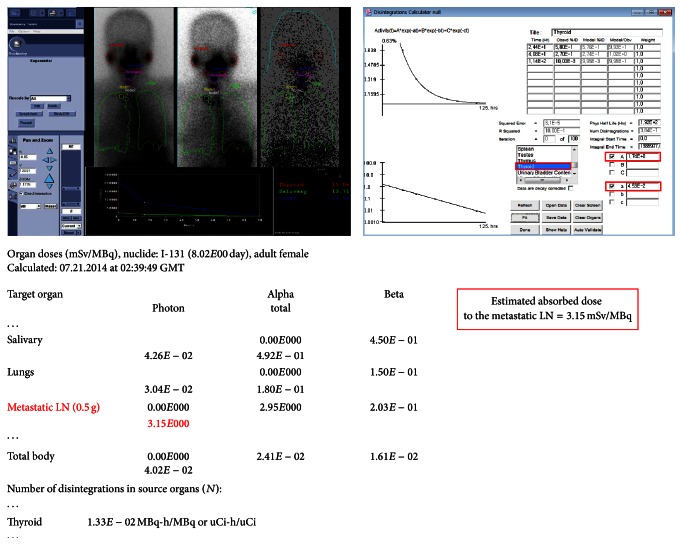
Dosimetric assessment with multiple whole-body and SPECT images using a commercialized program. The retention time of each organ is obtained by imaging data with the images and the estimated absorbed doses for the organs and lesions are calculated using the OLINDA program.

Currently, with the progresses being made in the field, dosimetric tools of both hardware and software are available in commercial nuclear medicine imaging systems, and their implementation is also quite simple (Figure 4). Therefore, intervention of a health physicist is no longer an essential prerequisite for dosimetry. With the advent of SPECT/CT technology, the 3-dimensional (3D) absorbed dose-rate distribution from the Monte Carlo based calculation can be used to obtain the absorbed dose of the target and surrounding tissues more accurately. The dosimetric data calculated with the 3D technology provides a more accurate personalized dose of I-131, which is not only high enough to have therapeutic effects on the target tissue but also low enough to avoid significant adverse effects to nontarget organs [23].

Although the dosimetric method is logically superior to the empirical fixed dose method for determining the therapeutic dose of I-131, a fixed empirical dose approach has been preferred in most centers due to its simplicity and easy performance [11]. Other reasons for the popularity of the fixed dose method might originate from additional costs, patient discomfort, and the stunning issue by the dosimetric study before I-131 treatment. In addition, superiority of the dosimetric method over the empirical method is not obvious due to the lack of comprehensive clinical trials designed to show the value of dosimetry for improving the therapy outcome [10, 23]. However, the dosimetric method can play important roles in I-131 treatment of differentiated thyroid cancer patients in unusual situations, such as distant thyroid cancer metastasis, renal insufficiency, or recombinant human thyroid stimulating hormone (TSH) application [10].

## 5. Radioiodine Treatment Coupled with Redifferentiation for Dedifferentiated Thyroid Cancer

Differentiated thyroid cancer has excellent prognosis compared to other malignancies, and this is partly related to the successful treatment of unresectable distant metastasis by therapeutic dose of I-131 administration. However, two-thirds of patients with distant metastases ultimately become radioiodine refractory disease [6, 7, 11, 20, 24–26]. The radioiodine refractory status is related to decreased expression of the NIS and diminished targeting of NIS to the membrane of cancer cells or both [26, 27]. Localized radioiodine refractory thyroid cancer can be treated with surgery, stereotactic external beam therapy, thermal or laser or alcohol ablation, chemoembolization, or radioembolization. However, these local therapeutic approaches cannot be applied in cases with metastatic lesions that cannot be approached or in cases with numerous metastatic lesions. Systemic chemotherapy or targeted therapy with tyrosine kinase inhibitors can be applied to systemic disease; however, it is not used routinely due to a low response rate and high rate of severe adverse effects. In addition, tyrosine kinase has cytostatic effects rather than cell-killing effects on cancer cells and it might not be easy to attain cure for the disease [26].

**Figure 5:**
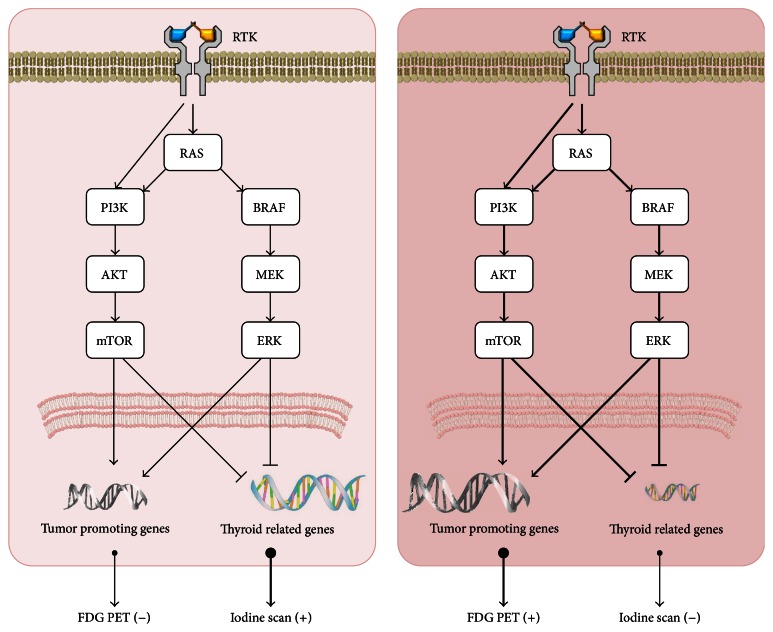
Signaling pathways related to iodine avidity or glucose avidity by thyroid cancers. Well-differentiated thyroid cancer has less iodine avidity and greater glucose avidity than normal thyroid follicular cell by activity of MAPK kinase and PI3K-AKT pathways, which inhibit the expression of thyroid related genes and reinforce the expression of tumor promoting genes. The pathways become more active in poorly differentiated thyroid cancer compared to well-differentiated thyroid cancer and eventually it loses the iodine avidity and acquires glucose avidity. RTK: receptor tyrosine kinase.

**Figure 6:**
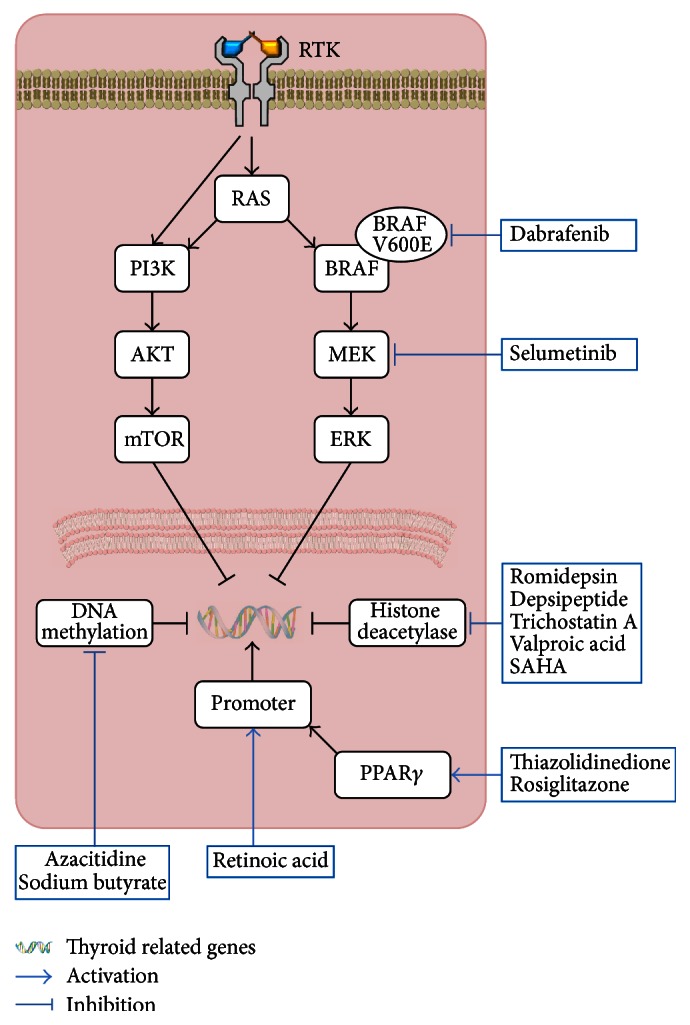
Strategies of pharmacologic redifferentiation of radioiodine refractory thyroid cancer by intervening genetic and epigenetic alterations and dysregulated signaling pathways related to the cancer.

Losing iodine avidity of differentiated thyroid cancer is related to genetic and epigenetic alterations and dysregulated signaling pathways, such as the MAPK and PI3K-AKT pathways, and research is currently on to identify compounds that are able to increase iodine uptake by enhancing NIS expression and migration to the plasma membrane [26, 28–30] (Figures 5 and 6). Several compounds, such as retinoic acid, PPAR*γ* agonists, HDAC inhibitors (valproic acid and carbamazepine), PI3K/AKT inhibitors, and MEK/ERK inhibitors, have been suggested to increase NIS expression and have resulted in increased iodine uptake in both* in vitro* and* in vivo* studies of thyroid cancers [28, 31].

The reinduction of NIS expression and iodine avidity in the radioiodine refractory cancer using redifferentiation therapy aids in curing the disease by applying I-131 treatment after the redifferentiation, similar to radioiodine therapy for unresectable metastatic differentiated thyroid cancer. To enhance radioiodine accumulation in radioiodine refractory thyroid cancer, retinoic acid has been used for more than 20 years to increase NIS expression. Although retinoic acid has the ability of increasing iodine uptake in the cancer, unfortunately, it is not used normally because the degree of iodine accumulation by the drug is limited and a clinical response to radioiodine treatment after retinoic acid administration was marginal or negligible [32]. Drugs acting on peroxisome proliferator-activated receptor *γ* (PPAR*γ*) also have the ability of increasing iodine uptake in preclinical studies. However, the degree of iodine uptake by the drugs was not considerable, and the drugs are not frequently used in clinics to redifferentiate radioiodine refractory thyroid cancer before radioiodine treatment due to the limited therapeutic response [26, 33].

Expression of thyroid related genes is known to be related to MAPK kinase and PI3K-AKT pathways, and certain types of thyroid cancers have abnormal pathway activation due to oncogenic mutations, such as the BRAF V600E mutation. Increased activity of these pathways suppresses expression and membrane targeting of NIS protein [29, 34, 35].

Glycosylation of the NIS protein, posttranslational event of binding carbohydrates to the protein, is important for proper localization of the transmembrane protein, which is essential for its function. Overexpression of PI3K decreases the glycosylation of the NIS protein and results in impairment of cellular iodine uptake [28]. The NIS gene expression and its migration to the proper location in a cell are differentially regulated based on the cell types, and therefore, certain drugs known to increase iodine uptake in one cell type will not work with equal potency or even reduce the iodine uptake in other cell types [28, 36]. Although certain radioiodine refractory thyroid cancers express NIS protein, the protein is predominantly localized in the cytoplasm and not in the plasma membrane, where it is not able to trap circulating iodine [37]. An understanding of the molecular mechanism for migration of NIS from the cytoplasm to the plasma membrane and methods to increase the translocation is needed for successful I-131 treatment of cancers with endogenous NIS expression. Unfortunately, the mechanism and methods might differ according to the subtypes of thyroid cancer, and therefore, the optimal strategy for enhancement of I-131 uptake should be tailored according to subtype [38].

Based on the redifferentiation effect of MAPK pathway inhibitor in preclinical studies, one clinical trial using the MEK inhibitor, selumetinib, revealed promising results of successful pharmacological redifferentiation of radioiodine refractory thyroid cancer [39].

## 6. Conclusions

Radioiodine- and NIS-based theranostics was used before coining the term “theranostics” and was successfully implemented as a representative model of personalized medicine for more than 70 years in the clinical field of differentiated thyroid cancer.

Personalized medicine using radioiodine theranostics can provide high therapeutic effects and avoid significant adverse effects through tailored therapeutic plans for individual patient, and rapid advancements in bio- and nuclear technologies can accelerate and broaden clinical application of the strategy for treating thyroid cancers with or without differentiation.

Preclinical studies for radioiodine theranostics are currently on to discover the hidden potential and the promising results are also being documented. Although inborn characteristics of limited universalization of personalized medicine may require time, the bench technology of radioiodine theranostics will be translated into clinical application as one of the tailored therapeutic approaches.
